# Genotypic evidence of benzimidazole resistance and population structure insights in zoonotic *Trichostrongylus colubriformis* from Thailand

**DOI:** 10.1007/s00436-026-08713-2

**Published:** 2026-06-15

**Authors:** Abigail Hui En Chan, Wallop Pakdee, Chanisara Kaenkaew, Punyanuch Kaewwongwattana, Sivapong Sungpradit, Urusa Thaenkham

**Affiliations:** 1 Laboratory of Helminth Biodiversity and Drug Development, Department of Helminthology, Faculty of Tropical Medicine, Mahidol University, Bangkok, Thailand; 2 Department of Pre-Clinic and Applied Animal Science, Faculty of Veterinary Science, Mahidol University, Nakhon Pathom, Thailand

**Keywords:** Benzimidazole, Genetic diversity, Resistance, *Trichostrongylus colubriformis*, Thailand

## Abstract

*Trichostrongylus* is a genus of zoonotic gastrointestinal nematode which is part of the strongylid group, where close contact with livestock is a risk factor for human infection. High levels of benzimidazole-resistant *Trichostrongylus* populations have been reported in Thailand, where the resistant allele frequency of *Trichostrongylus* populations were consistently higher than the resistant allele frequency of *Haemonchus.* Despite its zoonotic importance and the widespread use of benzimidazoles for gastrointestinal nematode infections in livestock, *Trichostrongylus* is often overshadowed by the more pathogenic *Haemonchus*. Here, we aim to assess the benzimidazole resistance status of *Trichostrongylus colubriformis* adults in Thailand, and investigate their genetic diversity and population structure. Using 60 *T. colubriformis* adults from Sukhothai and Satun, benzimidazole resistance was genotypically detected using the *b-tubulin* gene. Genetic diversity was investigated by amplification of the mitochondrial *COI* gene of representative specimens. Significant levels of benzimidazole resistance was detected, with the heterozygous (RS) genotype as the majority (81.7%). The population genetic structure revealed high genetic diversity of *T. colubriformis* populations and high genetic differentiation between the populations from Thailand and other countries, indicative of limited genetic exchange between populations. Despite significant levels of benzimidazole resistance in Thailand’s *T. colubriformis* population, the population structure, where limited genetic exchange and high genetic differentiation was observed between populations, suggests that locally targeted measures could be effective to curb anthelminthic resistance spread. However, additionally surveillance of this zoonotic nematode is advocated to sustainably safeguard animal and human health.

**Supplementary Information:**

The online version contains supplementary material available at 10.1007/s00436-026-08713-2.

## Introduction

*Trichostrongylus* is a genus of zoonotic gastrointestinal nematode within the strongylid group infecting livestock and humans (Bhat et al. 2023). They are widely distributed and prevalent in both tropical and temperate regions (Bhat et al. 2023). In livestock, *Trichostrongylus* infections can cause anemia and inflammation of the intestinal mucosa, leading to complications and death in young or weak animals (Bhat et al. 2023; Craig 2009). Consequently, significant losses in farm productivity result, due to decreased milk and meat yield. In humans, light infections cause mild symptoms such as abdominal pain or diarrhea, while heavy infections cause slight anemia and eosinophilia (Souza et al. 2013; Watthanakulpanich et al. 2013). In Thailand, *Trichostrongylus colubriformis* and *Trichostrongylus axei* are the most common *Trichostrongylus* species infecting humans. Although low prevalence in humans were previously reported, close contact with livestock is a risk factor for human infection (Phosuk et al. 2013, 2015; Wattanawong et al. 2021).

Anthelminthic resistance poses a global threat to animal health, human health, and the farming industry (Kapo et al. 2025; Sautier and Chiron 2023). Anthelminthic resistance contributes to the persistent high prevalence of helminthic infections in livestock, increasing the risk of zoonotic transmission. Benzimidazole resistance in *Trichostrongylus* was previously phenotypically evidenced from various countries, including Ethiopia, Poland, New Zealand, India, Italy, and Thailand (Chan et al. 2025a; Mickiewicz et al. 2020; Mukbel et al. 2025; Peña-Espinoza et al. 2014; Sontigun et al. 2025; Waghorn et al. 2014). Genotypic detection of the single nucleotide polymorphism (SNP) at the F200Y codon of the *b-tubulin* isotype 1 gene is used as a diagnostic marker and has proven to be more sensitive than phenotypic tests (Chan et al. 2025a; Silvestre and Humbert 2000). In Thailand, high levels of benzimidazole-resistant *Trichostrongylus* populations have been reported from goat farms in Ratchaburi and Nakhon Si Thammarat Provinces through genotype testing (Chan et al. 2025a; Sontigun et al. 2025). Moreover, the resistant allele frequency of *Trichostrongylus* populations were consistently higher than the resistant allele frequency of *Haemonchus* in both studies, possibly reflecting a neglect of *Trichostrongylus* monitoring due to its comparatively lower pathogenicity in livestock as compared to *Haemonchus* infections.

In Thailand, co-grazing farming practices, open concept farms, and the unregulated use of anthelminthic drugs can facilitate the transmission of helminthic infections and promote the spread of anthelminthic-resistant alleles. Extensive anthelminthic drug use, host movement, and environmental changes can influence the genetic diversity and population structure of nematode populations. Moreover, high genetic diversity and large effective population sizes of nematodes have been implicated in the rapid evolution of anthelminthic drug resistance (Brasil et al. 2012; Monteiro et al. 2025). Studies investigating *Haemonchus contortus* populations in livestock using mitochondrial genetic markers such as the mitochondrial cytochrome c oxidase 1 (*COI*) and NADH dehydrogenase 4 (*ND4*) genes have revealed high genetic diversity and low genetic differentiation between populations, likely reflecting extensive host movement (Chan et al. 2025b; Hussain et al. 2014; Parvin et al. 2024; Pitaksakulrat et al. 2021a). Population expansion was also evidenced, suggesting positive selection for advantageous mutations to ensure parasite survival and reproduction under drug pressure. For *Trichostrongylus*, Archie and Ezenwa (2010) revealed high genetic diversity and low genetic differentiation between *T. axei* populations from different sympatric hosts in the United States of America, suggesting population expansion and gene flow (Archie and Ezenwa 2011).

Despite its zoonotic importance and the widespread use of benzimidazoles in livestock, *Trichostrongylus* is often overshadowed by the more pathogenic *Haemonchus*. Furthermore, as *Trichostrongylus* is zoonotic, the risk of anthelminthic resistance spread to humans emphasizes the importance of surveillance. Here, we aim to assess the benzimidazole resistance status of *T. colubriformis* adults in Thailand, providing a genotypic characterization of resistance patterns in two populations (Sukhothai and Satun provinces in lower northern and southern Thailand, respectively). The genetic diversity and population structure of *T. colubriformis* was also investigated, to infer evolutionary pressures which may be acting on these populations. This information can offer insights regarding resistance patterns and strategies for the sustainable control of *Trichostrongylus* and mitigate resistance spread in Thailand.

## Materials and methods

### *Trichostrongylus* specimen collection and identification

**Fig. 1:**
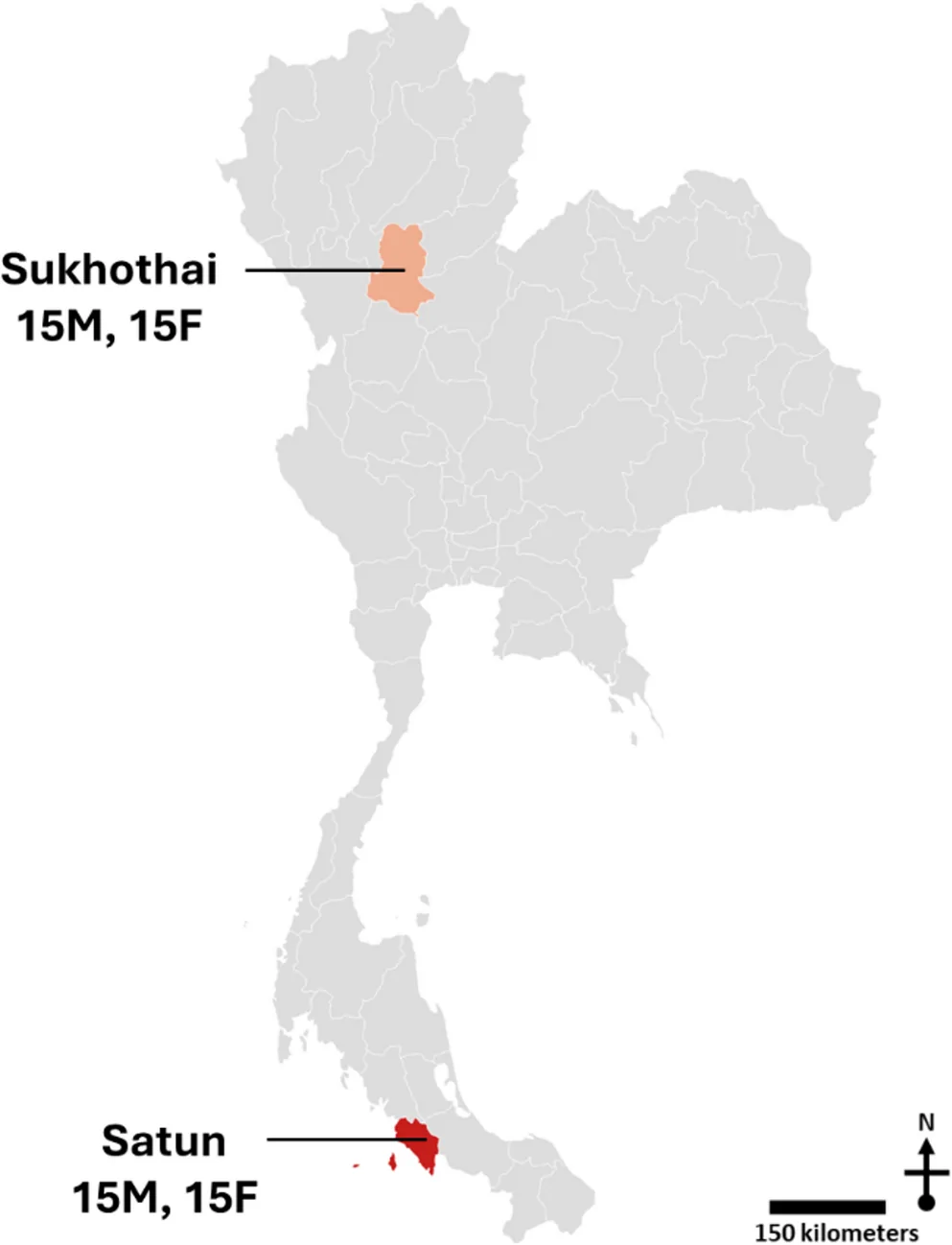
Map showing the locations where adult *T. colubriformis* was obtained. The numbers represent the number of males (M) and females (F) sampled

Male and female *Trichostrongylus* adults were isolated from the small intestines of goats obtained from slaughterhouses in Sukhothai and Satun (Fig. 1). Briefly, the small intestines were dissected, and the contents were allowed to sediment in water. The sediment was observed under a stereomicroscope (Olympus SZ51) to isolate the *Trichostrongylus* adults. The collected specimens were washed in normal saline, counted, and observed under an inverted compound microscope (ZEISS Primovert) for morphological identification. A total of 60 *Trichostrongylus* were selected, comprising of 30 specimens per province. The specimens were individually kept in 70% ethanol at − 20 °C for preservation prior to downstream molecular work.

### Genotypic detection of benzimidazole resistance at the F200Y codon

The 60 specimens were washed thoroughly with sterile distilled water and transferred into 1.5 ml Eppendorf tubes. Total genomic DNA was isolated from each specimen using the Geneaid gDNA Mini Kit (Geneaid Biotech Ltd, Taipei, Taiwan) following the manufacturer’s recommendations.

Using allele-specific PCR, genotypic detection of the F200Y mutation in the *b-tubulin* isotype 1 gene of *T. colubriformis* was performed. The allele-specific PCR was conducted using a T100™ thermocycler (Bio-Rad, USA) in a 20 µl PCR reaction mix which contained 10 µl of 2X iTaqTM master mix (iNtRON Biotechnology, Gyeonggi, South Korea), 10 µM of each primer (Pc1: 5’ - GGAACAATGGATTCCGTTCG − 3’ and Pc4: 5’ - ATACAGAGCTTCGTTATCGATGCAGA − 3’ for the susceptible allele; and Pc2: 5’ - GGGAATCGGAGGCAAGTCGT- 3’ and Pc3: 5’ - CTGGTAGAGAATACCGATGAAACATA- 3’ for the resistant allele), and 1 ng/µl of gDNA. The primers and PCR conditions follow Silvestre and Humbert (2000) (Silvestre and Humbert 2000). Amplicons (550 bp for the susceptible allele and 250 bp for the resistant allele) were visualized under an UV light after electrophoresis in a 1.5% agarose gel gel stained with SYBR™ Safe (Thermo Fisher Scientific, MA, USA). One representative specimen was sent for sequencing by FastNGS (Tsingke Biotech, Beijing, China) to confirm the presence of the SNP (TTC to TAC) at the F200Y codon.

For each specimen, the genotype was determined based on the presence or absence of the appropriate band size for each allele. The percentage for each genotype (SS: homozygous susceptible, RR: homozygous resistant, and RS: heterozygous) was calculated. The susceptible and resistant allele frequencies were calculated by taking the number of each allele divided by the total number of alleles in the population.

### Genetic variation of *Trichostrongylus* using the *COI* gene

Following genotypic detection of benzimidazole resistance, 30 representative *T. colubriformis* specimens (15 from each province) that contained the resistant allele were selected for amplification of the partial mitochondrial *COI* gene. Primers (LCO1490: 5’ - GGTCAACAAATCATAAAGATATTGG − 3’ and HCO2198: 5′-TAAACTTCAGGGTGACCAAAAAATCA − 3′) and PCR amplification followed the protocol of Folmer et al. (1994) (Folmer et al. 1994). Amplification was conducted in a final volume of 30 µl, which contained 15 µl of 2X iTaq™ master mix (iNtRON Biotechnology, Gyeonggi, South Korea), 25 µM of each primer, and 1 ng/µl of gDNA. The 710 bp amplicons were visualized under UV light after electrophoresis in a 1% agarose gel stained with SYBR™ Safe (Thermo Fisher Scientific, MA, USA) and sent for FastNGS sequencing (Tsingke Biotech, Beijing, China).

The electropherograms were checked using Bioedit 7.0 (Hall 1999) and aligned in ClustalX 2.1 (Thompson et al. 2002) with reference *T. colubriformis* sequences from the NCBI database. *Trichostrongylus axei* was used as the outgroup. Phylogenetic analysis was conducted in MEGA X (Kumar et al. 2018), using the maximum likelihood (ML) method. The best-fit nucleotide substitution model with 1000 bootstrap iterations was used for the ML phylogeny. Pairwise genetic distances were calculated in MEGA X (Kumar et al. 2018) and phylogenies were visualized and labelled using FigTree 1.3.1 (Rambaut 2010).

Along with the reference sequences, genetic diversity indices such as the number of haplotypes, haplotype diversity (H_d_), nucleotide diversity (π), neutrality tests (Fu and Li’s F statistic and Tajima’s D), non-synonymous (Ka) and synonymous (Ks) substitution, and genetic differentiation (F_ST_) were calculated in DnaSP version 6 (Rozas et al. 2017). A median-joining haplotype network was constructed using PopART version 1.7 (Leigh and Bryant 2015).

## Results

### Benzimidazole resistance status of *Trichostrongylus*

**Fig. 2:**
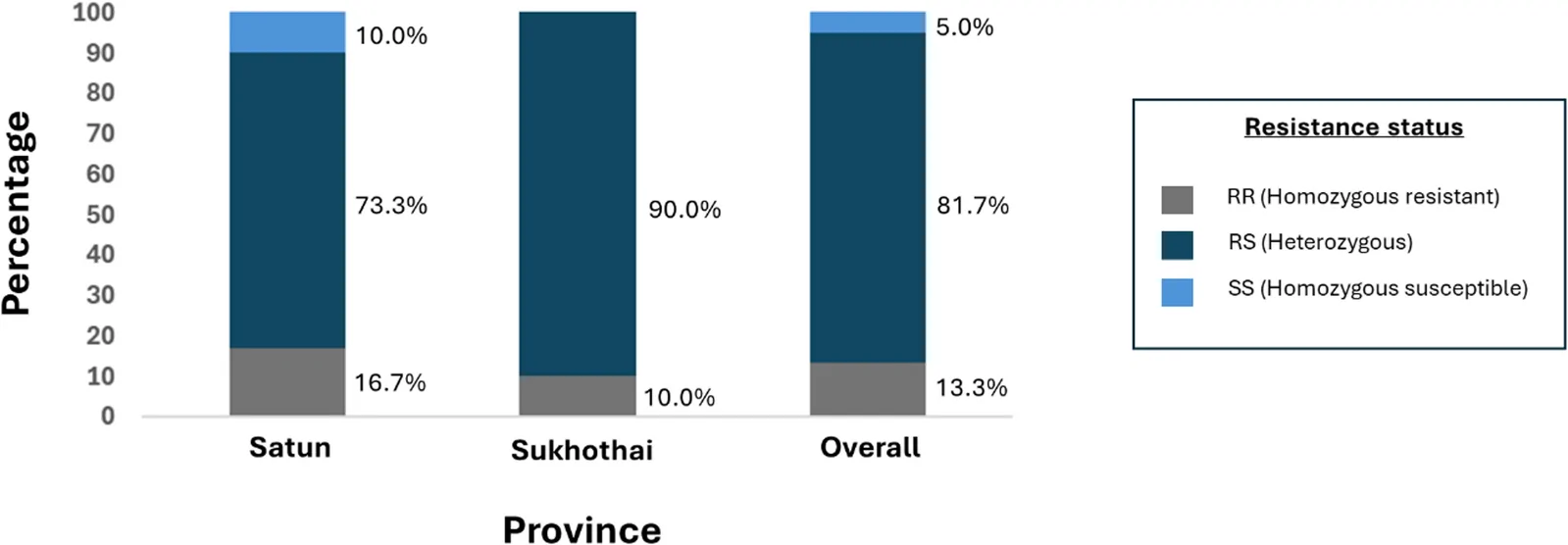
Proportion of benzimidazole genotypes obtained from the two *Trichostrongylus* populations in Thailand

**Fig. 3:**
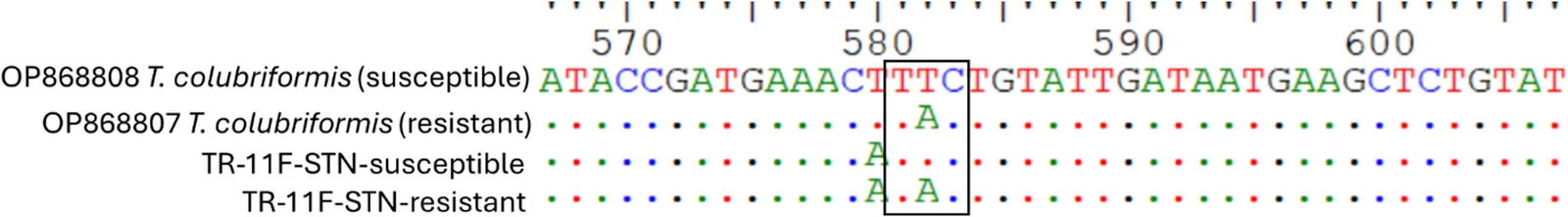
Confirmation of the TTC to TAC mutation at the F200Y codon of the *b-tubulin* isotype 1 gene

From the 60 *Trichostrongylus* specimens, the heterozygous (RS) genotype was the majority at 81.7%, followed by the homozygous resistant (RR) genotype at 13.3% and the homozygous susceptible (SS) genotype at 5.0%. The percentage of genotypes obtained from the two populations is presented in Fig. 2, while the genotype for each specimen is in Supplementary File 1. A higher proportion of the RS genotype was present in Sukhothai than Satun, while no SS genotype was found in Sukhothai. The overall frequency of the susceptible and resistant alleles was 0.46 and 0.54, respectively (Table 1), with similar values obtained for both provinces. Evidence of the TTC to TAC mutation at the F200Y codon, indicative of benzimidazole resistance is depicted in Fig. 3, while an example of the amplicons obtained after gel electrophoresis are in Supplementary File 2.

**Table 1:** Benzimidazole susceptible and resistant allele frequencies

Province	No. of specimens	Resistant allele frequency	Susceptible allele frequency
Satun	30	0.53	0.47
Sukhothai	30	0.55	0.45
Overall	60	0.54	0.46

### Genetic diversity of *Trichostrongylus colubriformis*

**Fig. 4:**
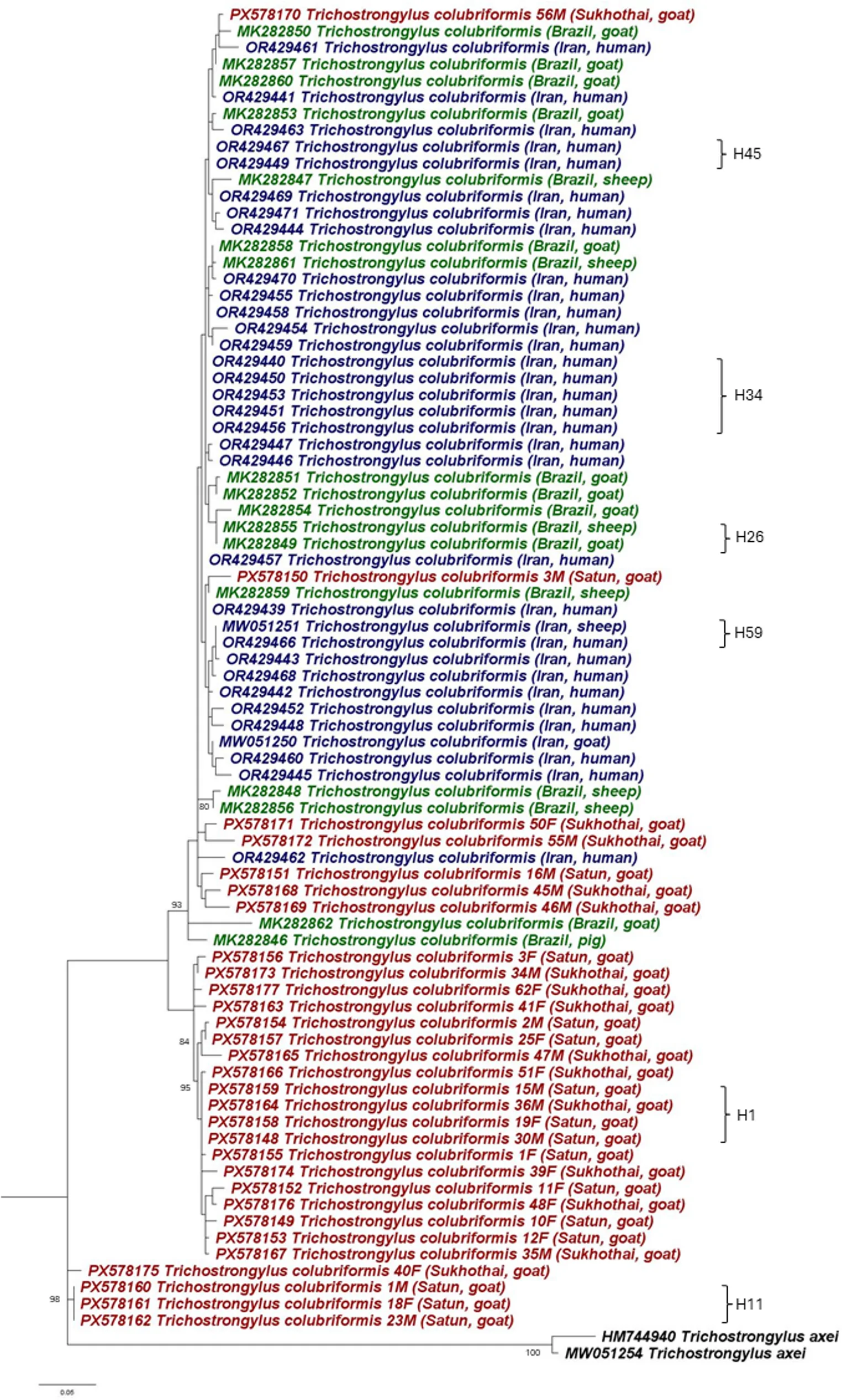
Maximum likelihood phylogeny of *Trichostrongylus* using the partial mitochondrial *COI* gene. Only bootstrap values above 70 are shown. The sequences generated in this study from Thailand are coded in red, while the sequences obtained from the NCBI database are in blue for Iran and green for Brazil. The host and locality are labeled in parentheses. The haplotypes that contain more than one sequence are indicated in the square brackets

**Fig. 5:**
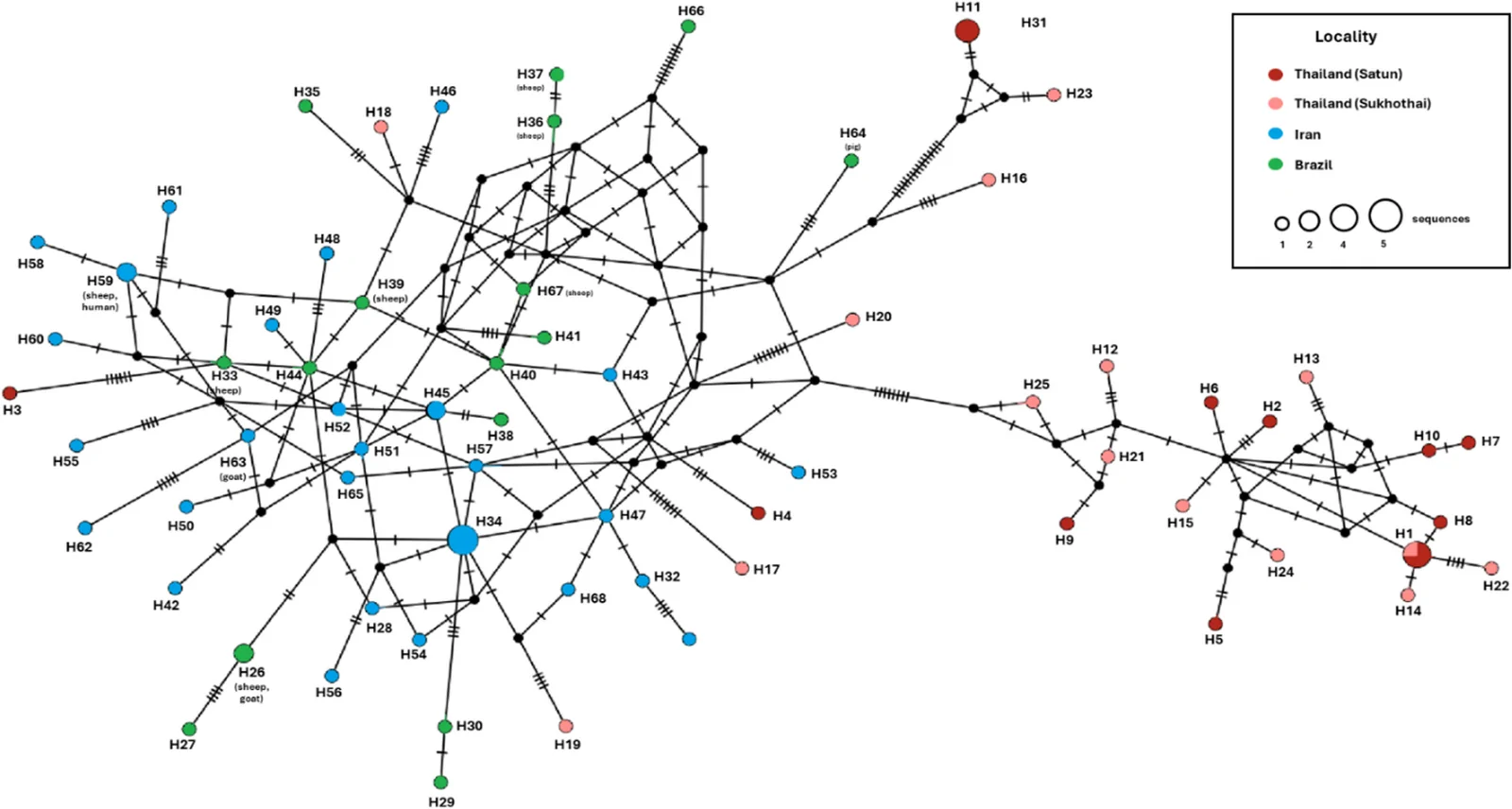
Median-joining haplotype network of *T. colubriformis* using the partial mitochondrial *COI* gene (384 bp). Each circle represents a haplotype, where the number of sequences for each haplotype is proportional to the size of the circle. The dashes separating the circles indicate the number of mutational changes. Hosts that are not of the majority from each locality are indicated in parentheses. Goat is the majority host for Thailand and Brazil, while human is the majority host for Iran

Using 30 representative *T. colubriformis* sequences, along with 33 and 17 other sequences from Iran and Brazil, respectively, the genetic diversity and population genetic structure was investigated. The ML phylogeny with the partial mitochondrial *COI* gene revealed differentiation of Thailand’s *T. colubriformis* population, where majority of specimens are genetically distinct from Iran and Brazil (Fig. 4). Of the 30 sequences, seven were clustered in the clade together with sequences from Iran and Brazil. Intraspecies genetic distances within Thailand was the highest at 4%, followed by 2% and 1% for Iran and Brazil, respectively. No genetic distinction was found between Sukhothai and Satun. Similarly, the haplotype network revealed distinction between Thailand’s population and the populations from Iran and Brazil (Fig. 5). High levels of genetic differentiation were observed between the populations, with F_ST_ values of 0.420 between Thailand and Iran and 0.363 between Thailand and Brazil. Low level of genetic differentiation (F_ST_ value of 0.067) was obtained between Iran and Brazil, while no genetic differentiation was observed between the populations from Sukhothai and Satun provinces.

The largest haplotype obtained from the 384 bp sequences was H34, comprising of five sequences from humans in Iran. Among the sequences from Thailand, the largest haplotype (H1) contained four sequences, three from Satun and one from Sukhothai. Of the 68 haplotypes obtained overall, only 6 haplotypes contained more than one sequence, demonstrating high genetic diversity. The diversity indices presented in Table 2 showed an overall haplotype diversity of 0.993 and 0.979 for Thailand’s sequences, while the nucleotide diversity was 0.032 (overall) and 0.037 (Thailand). The neutrality test values of Fu and Li’s F statistics and Tajima’s D were − 2.941 and − 1.504 overall, respectively, suggesting population expansion. With a Ka: Ks ratio of more than 1, the direction of selection was positive, suggesting the selection of advantageous mutations.

**Table 2:** Diversity and neutrality indices of the *T. colubriformis* populations from Thailand, Iran, and Brazil using the partial mitochondrial *COI* gene

Country	No. of sequences	No. of haplotypes	Haplotype diversity (H_d_)	Nucleotide diversity (π)	Fu and Li’s F statistic	Tajima’s D	Ka: Ks ratio
Thailand	30	25	0.979	0.037	-1.186	-0.894	> 1
Iran	33	27	0.977	0.013	-2.638	-1.361	> 1
Brazil	17	16	0.993	0.021	-1.514	-1.418	> 1
Overall	80	68	0.993	0.032	-2.941	-1.504	> 1

## Discussion

Using *T. colubriformis* adults from representative provinces in the lower northern and southern regions of Thailand, we found significant levels of benzimidazole resistance in both populations, especially of the heterozygous genotype. High genetic diversity was observed between populations from Thailand, Iran, and Brazil, while no genetic differentiation was present between the Thai populations (Sukhothai and Satun).

Firstly, a combined proportion of 86.7% was observed for both the RS and RR genotypes, while the SS genotype only consisted of 13.3% of the population, indicating significant levels of benzimidazole resistance. Although the combined resistant allele frequency was 0.54, the frequency observed in this study was higher than that was observed for adult *H. contortus* (0.44) sampled from six provinces in Thailand (Chan et al. 2025b). Sontigun et al. (2025) also reported a high resistant allele frequency (0.91) of *T. colubriformis* larvae populations across six farms in Nakhon Si Thammarat province, which was higher than the *H. contortus* resistant allele frequency (0.78) (Sontigun et al. 2025). Similarly, a survey of 30 goat farms in Ratchaburi province using pooled larval samples revealed a higher resistant allele frequency for *Trichostrongylus* (0.74) as compared to *Haemonchus* (0.65), with *Trichostrongylus*-resistant populations present in 100% of the surveyed farms (Chan et al. 2025a). As the treatment for *Trichostrongylus* and *Haemonchus* infections in livestock is the same and they are often found infecting the same host, the selective pressure of resistance in *H. contortus* can also drive resistance in *T. colubriformis* (Fissiha and Kinde 2021; Klauck et al. 2014). In Thailand, elevated resistance levels are no surprise, as goats are treated frequently with anthelminthics (Department of Livestock Development 2017; Pitaksakulrat et al. 2021b). Moreover, as *T. colubriformis* is zoonotic, the potential for resistant allele spread to humans is highly plausible with close animal contact. This phenomenon has been observed in other zoonotic helminths such as *Fasciola hepatica*, with triclabendazole treatment failure reported in humans following its widespread use in livestock (Caravedo and Cabada 2020; Kelley et al. 2016; Morales et al. 2021; Winkelhagen et al. 2012). Thus, the spread of resistant alleles in a zoonotic nematode represents a significant threat to both animal and human health.

Secondly, the population structure revealed high genetic diversity and no genetic differentiation between the Thai populations. Gene flow and population expansion are evident, with genetic material freely exchanging between the two populations in Thailand, likely due to frequent goat movement between farms and provinces (Chintrakulchai et al. 2017). Similar patterns were seen in Thailand’s *H. contortus* populations from previous studies, further supporting the hypothesis of host movement, where goats are frequently transported from one province to another within the country (Chan et al. 2025b; Parvin et al. 2024). High genetic variability among *T. colubriformis* from sheep and goats in Brazil was also reported by Monteiro et al. (2025), as compared with other gastrointestinal nematodes sampled (Monteiro et al. 2025). Here, the selection for beneficial mutations is inferred by the positive selection pressure, which allows for advantageous traits to be selected within populations. Over time, these mutations increase in frequency and become fixed. Consequently, parasite populations with advantageous traits can survive and propagate, increasing the chances of survival for populations with the benzimidazole-resistant allele (Maingi et al. 1990). Thus, the spread of resistant alleles between Thailand’s *T. colubriformis* populations may be facilitated by host movement and selective pressures as observed in the genetic structure.

Comparing between countries, high genetic differentiation was observed between Thailand’s *T. colubriformis* population with those from either Iran or Brazil. Contrarily, low genetic differentiation was observed between the worm populations in Iran and Brazil. The results thus indicate limited genetic exchange of *T. colubriformis* occurring between Thailand’s population and other countries, reflecting the lack of host movement between them. The limited gene flow suggests that benzimidazole resistance in *T. colubriformis* populations may have emerged locally, rather than through cross-boundary genetic exchange. Consequently, the potential transboundary spread of resistance alleles can be slowed if appropriate measures are put in place (e.g., controlling cross-country goat import and export), in contrast to *H. contortus*, where benzimidazole resistance is widespread (Chandra et al. 2015; Dey et al. 2020; Kapo et al. 2024; Potârniche et al. 2021; Santos et al. 2014). Currently, benzimidazole-resistant *Trichostrongylus* populations have been phenotypically reported from some countries in Europe, Asia, and Africa, with genotypic testing conducted only in Thailand and Mozambique (Chan et al. 2025a; Guinda et al. 2025; Mickiewicz et al. 2020; Mukbel et al. 2025; Peña -Espinoza et al. 2014; Sontigun et al. 2025; Waghorn et al. 2014). However, the limited data on the anthelminthic resistance of *Trichostrongylus* warrants caution in the intepretation of the actual resistance status, where it may be underestimated. Thus, it highlights a knowledge gap that should be addressed to fully understand the threat and risk posed by anthelminthic resistance of this zoonotic nematode.

The limitations of this study include the small population and sample size, where only two provinces were surveyed. Nonetheless, the study provides the first genotypic evidence of benzimidazole resistance in *Trichostrongylus* along with a unique population structure when comparing with popualtions from Brazil and Iran, allowing a deeper look at evolutionary pressures which may shape their resistance patterns and population structure. Also, information regarding the anthelminthic drug history was not obtained. Finally, benzimidazole resistance detection was based on the mutation at the F200Y codon, while the F167Y and E198A were not tested for,.

## Conclusion

Through the first genotypic detection of benzimidazole resistance in *T. colubriformis* adults in Thailand and examining their genetic diversity, we identified high levels of benzimidazole resistance and genetic diversity. These findings are indicative of the selection for beneficial mutations within the country. The presence of high genetic differentiation between Thailand’s *T. colubriformis* population and other countries reflects limited host movement across borders, suggesting that locally targeted mitigation measures could be effective to curb anthelminthic resistance spread. With appropriate control strategies, benzimidazole resistance in Thailand’s *T. colubriformis* populations can be sustainably managed. However, the low frequencies of susceptible homozygotes may be indicative of a greater issue if appropriate control strategies are not put in place. Thus, greater surveillance of this under studied zoonotic nematode is advocated, especially in neighbouring countries, alongside the detection of other polymorphisms to gain a complete picture of the resistance status and patterns of *Trichostrongylus*.

## Supplementary Information

Supplementary Material 1.

Supplementary Material 2.

## Data Availability

All data generated are included in the published article and its additional information files. The *COI* gene sequences of representative sequences are in the NCBI database under the accession numbers PX578148 to PX578177. The b-tubulin gene sequences of the susceptible and resistant alleles are in the NCBI database under the accession numbers PZ338754 to PZ338755.

## References

[CR1] Archie E, Ezenwa V (2011) Population genetic structure and history of a generalist parasite infecting multiple sympatric host species. Int J Parasitol 41:89–98. 10.1016/j.ijpara.2010.07.014 20828576 10.1016/j.ijpara.2010.07.014

[CR2] Bhat AH, Tak H, Malik IM, Ganai BA, Zehbi N (2023) Trichostrongylosis: a zoonotic disease of small ruminants. J Helminthol 97:1–11. 10.1017/S0022149X2300007X 10.1017/S0022149X2300007X 36810301

[CR3] Brasil B, Nunes R, Bastianetto E, Drummond M, Carvalho D, Leite R, Molento M, Oliveira D (2012) Genetic diversity patterns of *Haemonchus placei* and *Haemonchus contortus* populations isolated from domestic ruminants in Brazil. Int J Parasitol 42:469–479. 10.1016/j.ijpara.2012.03.003 22787588 10.1016/j.ijpara.2012.03.003

[CR4] Caravedo M, Cabada M (2020) Human fascioliasis: current epidemiological status and strategies for diagnosis, treatment, and control. Res Rep Trop Med 11:149–158. 10.2147/RRTM.S237461 33273878 10.2147/RRTM.S237461 PMC7705270

[CR5] Chan AHE, Kaenkaew C, Pakdee W, Sungpradit S, Thaenkham U (2025a) Emergence of dual drug-resistant strongylids in goats: first phenotypic and genotypic evidence from Ratchaburi Province, central Thailand. BMC Vet Res 21:245. 10.1186/s12917-025-04700-4 40181391 10.1186/s12917-025-04700-4 PMC11969839

[CR6] Chan AHE, Thaenkham U, Kaenkaew C, Pakdee W, Sungpradit S (2025b) Hybridization, high genetic diversity, and dual drug resistance in *Haemonchus* populations infecting goats in Thailand. BMC Vet Res 21:679. 10.1186/s12917-025-05133-9 41272644 10.1186/s12917-025-05133-9 PMC12639715

[CR7] Chandra S, Prasad A, Yadav N, Latchumikanthan A, Rakesh R, Praveen K, Khobra V, Subramani K, Misri J, Sankar M (2015) Status of benzimidazole resistance in *Haemonchus contortus* of goats from different geographic regions of Uttar Pradesh, India. Vet Parasitol 208:263–267. 10.1016/j.vetpar.2015.01.005 25680901 10.1016/j.vetpar.2015.01.005

[CR8] Chintrakulchai P, Vuttichai S, Wiratsudakul A (2017) Goat movement network analysis and its implications for caprine brucellosis propagation in Nonthaburi Province, Thailand. Asian Pac J Trop Dis 7:477–481. 10.12980/apjtd.7.2017D7-85

[CR9] Craig T (2009) Helminth parasites of the ruminant gastrointestinal tract. In: Anderson DE, Rings DM (eds) Food animal practice, 5th edn. Elsevier, Amsterdam, pp 78–91

[CR10] Department of Livestock Development (2017) Annual Report 2016. Department of Livestock Development, Ministry of Agricultural and Cooperatives, Bangkok, Thailand

[CR11] Dey AR, Begum N, Anisuzzaman MA, Alam MZ (2020) Multiple anthelmintic resistance in gastrointestinal nematodes of small ruminants in Bangladesh. Parasitol Int 77:102105. 10.1016/j.parint.2020.102105 32179135 10.1016/j.parint.2020.102105

[CR12] Fissiha W, Kinde M (2021) Anthelmintic resistance and its mechanism: a review. Infect Drug Resist 14:5403–5410. 10.2147/IDR.S332378 34938088 10.2147/IDR.S332378 PMC8687516

[CR13] Folmer O, Black M, Hoeh W, Lutz R, Vrijenhoek R (1994) DNA primers for amplification of mitochondrial cytochrome c oxidase subunit I from diverse metazoan invertebrates. Mol Mar Biol Biotechnol 3:294–299 7881515

[CR14] Guinda E, Afonso S, Fiedler S, Morgan E, Ramünke S, Borchert M, Atanásio A, Capece B, Krücken J, von Samson-Himmelstjerna G (2025) Efficacy of fenbendazole against gastrointestinal nematodes in naturally infected goats in Maputo Province, Mozambique using in vivo, in vitro and molecular assessment. Int J Parasitol Drugs Drug Resist 27:100572. 10.1016/j.ijpddr.2024.100572 39671856 10.1016/j.ijpddr.2024.100572 PMC11697842

[CR15] Hall TA (1999) BioEdit: A user-friendly biological sequence alignment editor and analysis program for windows 95/98/NT. Nucleic Acids Symposium Series 41:95–98

[CR16] Hussain T, Periasamy K, Nadeem A, Babar M, Pichler R, Diallo A (2014) Sympatric species distribution, genetic diversity and population structure of *Haemonchus* isolates from domestic ruminants in Pakistan. Vet Parasitol 206:188–199. 10.1016/j.vetpar.2014.10.026 25468018 10.1016/j.vetpar.2014.10.026

[CR17] Kapo N, Omeragić J, Goletić S, Šabić E, Softić A, Smajlović A, Mujezinović I, Škapur V, Goletić T (2024) First report of benzimidazole resistance in field population of *Haemonchus contortus* from sheep, goats and cattle in Bosnia and Herzegovina. Pathogens 13:77. 10.3390/pathogens13010077 38251384 10.3390/pathogens13010077 PMC10818805

[CR18] Kapo N, Softić A, Goletić T, Goletić S, Cvetkovikj A, Omeragić J (2025) Anthelmintic resistance in livestock farming: challenges and perceptions of farmers and veterinarians. Pathogens 114:649. 10.3390/pathogens14070649 10.3390/pathogens14070649 PMC12298476 40732697

[CR19] Kelley J, Elliott T, Beddoe T, Anderson G, Skuce P, Spithill T (2016) Current threat of triclabendazole resistance in *Fasciola hepatica*. Trends Parasitol 32:458–469. 10.1016/j.pt.2016.03.002 27049013 10.1016/j.pt.2016.03.002

[CR20] Klauck V, Pazinato R, Lopes L, Cucco D, Lima H, Volpato A, Radavelli W, Stefani L, Silva A (2014) *Trichostrongylus* and *Haemonchus* anthelmintic resistance in naturally infected sheep from southern Brazil. Acad Bras Cienc 86:777–784 30514017

[CR21] Kumar S, Stecher G, Li M, Knyaz C, Tamura K (2018) MEGA X: Molecular Evolutionary Genetics Analysis across Computing Platforms. Mol Biol Evol 35:1547–1549. 10.1093/molbev/msy096 29722887 10.1093/molbev/msy096 PMC5967553

[CR22] Leigh JW, Bryant D (2015) PopART: Full-feature software for haplotype network construction. Methods Ecol Evol 6:1110–1116

[CR23] Maingi N, Scott ME, Prichard RK (1990) Effect of selection pressure for thiabendazole resistance on fitness of *Haemonchus contortus* in sheep. Parasitology 100:327–335. 10.1017/S0031182000061345 2345665 10.1017/s0031182000061345

[CR24] Mickiewicz M, Czopowicz M, Kawecka-Grochocka K, Moroz A, Szaluś-Jordanow O, Várady M, Königová A, Spinu M, Górski P, Bagnicka E, Kaba J (2020) The first report of multidrug resistance in gastrointestinal nematodes in goat population in Poland. BMC Vet Res 16:270. 10.1186/s12917-020-02501-5 32746828 10.1186/s12917-020-02501-5 PMC7398340

[CR25] Monteiro K, Calegar D, Coronato-Nunes B, Santos J, Reis E, Bacelar P, Rossi M, Boia M, Monteiro F, Carvalho-Costa F, Jaeger L (2025) Molecular characterization of Strongylida infecting goats and sheep in northeastern Brazil using cytochrome-c oxidase subunit partial sequencing. Vet Parasitol Reg Stud Rep 61:101274. 10.1016/j.vprsr.2025.101274 10.1016/j.vprsr.2025.101274 40398990

[CR26] Morales M, Tanabe M, White C, Lopez M, Bascope R, Cabada M (2021) Triclabendazole treatment failure for *Fasciola hepatica* infection among preschool and school-age children, Cusco, Peru. Emerg Infect Dis 26:7. 10.3201/eid2707.203900 10.3201/eid2707.203900 PMC8237897 34152949

[CR27] Mukbel R, Kreishan A, Hammad H, Al-Sabi M (2025) First report of anthelmintic resistance among sheep in six farms from three governorates in Jordan. Vet Parasitol 57:101171. 10.1016/j.vprsr.2024.101171 10.1016/j.vprsr.2024.101171 39855859

[CR28] Parvin S, Dey AR, Shohana NN, Anisuzzaman Talukder MH, Alam MZ (2024) *Haemonchus contortus*, an obligatory haematophagus worm infection in small ruminants: Population genetics and genetic diversity. Saudi J Biol Sci 31:104030. 10.1016/j.sjbs.2024.104030 38854893 10.1016/j.sjbs.2024.104030 PMC11157266

[CR29] Peña -Espinoza M, Thamsborg S, Demeler J, Enemark H (2014) Field efficacy of four anthelmintics and confirmation of drug-resistant nematodes by controlled efficacy test and pyrosequencing on a sheep and goat farm in Denmark. Vet Parasitol 206:208–215. 10.1016/j.vetpar.2014.10.017 25468020 10.1016/j.vetpar.2014.10.017

[CR31] Phosuk I, Intapan PM, Sanpool O, Janwan P, Thanchomnang T, Sawanyawisuth K, Morakote N, Maleewong W (2013) Molecular evidence of *Trichostrongylus colubriformis* and *Trichostrongylus axei* infections in humans from Thailand and Lao PDR. Am J Trop Med Hyg 89:376–379. 10.4269/ajtmh.13-0113 10.4269/ajtmh.13-0113 PMC3741265 23798585

[CR30] Phosuk I, Intapan MP, Prasongdee KT, Changtrakul Y, Sanpool O, Janwan P, Maleewong W (2015) Human trichostrongylosis: a hospital case series. Southeast Asian J Trop Med Public Health 46:191–197 26513921

[CR32] Pitaksakulrat O, Chaiyasaeng M, Artchayasawat A, Eamudomkarn C, Boonmars T, Kopolrat KY, Prasopdee S, Petney T, Blair D, Sithithaworn P (2021a) Genetic diversity and population structure of *Haemonchus contortus* in goats from Thailand. Infect Genet Evol 95:105021. 10.1016/j.meegid.2021.105021 34363986 10.1016/j.meegid.2021.105021

[CR33] Pitaksakulrat O, Chaiyasaeng M, Artchayasawat A, Eamudomkarn C, Thongsahuan S, Boonmars T (2021b) The first molecular identification of benzimidazole resistance in *Haemonchus contortus* from goats in Thailand. Vet World 14:764–768. 10.14202/vetworld.2021.764-768 33935425 10.14202/vetworld.2021.764-768 PMC8076480

[CR34] Potârniche AV, Mickiewicz M, Olah D, Cerbu C, Spînu M, Hari A, Györke A, Moroz A, Czopowicz M, Várady M, Kaba J (2021) First report of anthelmintic resistance in gastrointestinal nematodes in goats in Romania. Animals 11:2761. 10.3390/ani11102761 34679782 10.3390/ani11102761 PMC8532838

[CR35] Rambaut A (2010) FigTree v1.3.1. Institute of Evolutionary Biology, University of Edinburgh, Edinburgh. http://tree.bio.ed.ac.uk/software/figtree/

[CR36] Rozas J, Ferrer-Mata A, Sánchez-DelBarrio JC, Guirao-Rico S, Librado P, Ramos-Onsins SE, Sánchez-Gracia A (2017) DnaSP 6: DNA Sequence Polymorphism Analysis of Large Data Sets. Mol Biol Evol 34:3299–3302. 10.1093/molbev/msx248 29029172 10.1093/molbev/msx248

[CR37] Santos J, Monteiro J, Ribeiro W, Macedo I, Camurca-Vasconcelos A, Vieira L, Bevilaqua C (2014) Identification and quantification of benzimidazole resistance polymorphisms in *Haemonchus contortus* isolated in Northeastern Brazil. Vet Parasitol 199:160–164. 10.1016/j.vetpar.2013.11.006 24295955 10.1016/j.vetpar.2013.11.006

[CR38] Sautier M, Chiron P (2023) Challenges and opportunities for reducing anthelmintic use in ruminant livestock systems: insights from a sheep farmer survey in France. Prev Vet Med 221:106078. 10.1016/j.prevetmed.2023.106078 38039933 10.1016/j.prevetmed.2023.106078

[CR39] Silvestre A, Humbert JF (2000) A molecular tool for species identification and benzimidazole resistance diagnosis in larval communities of small ruminant parasites. Exp Parasitol 95:271–276. 10.1006/expr.2000.4542 11038310 10.1006/expr.2000.4542

[CR40] Sontigun N, Sansamur C, Klong-Klaew T, Mektrirat R, Kaewthamasorn M, Fungwithaya P (2025) Field-based and molecular evaluation of anthelmintic resistance in gastrointestinal strongyle nematodes of meat goats in Southern Thailand. Vet World 18:2467–2478. 10.14202/vetworld.2025.2467-2478 41064838 10.14202/vetworld.2025.2467-2478 PMC12501572

[CR41] Souza R, Souza J, Menezes J, Alcântara L, Soares N, Teixeira M (2013) Human infection by *Trichostrongylus* spp. in residents of urban areas of Salvador city, Bahia, Brazil. Biomedica 33:439–445. 10.7705/biomedica.v33i3.770 24652180 10.7705/biomedica.v33i3.770

[CR42] Thompson JD, Gibson TJ, Higgins DG (2002) Multiple sequence alignment using ClustalW and ClustalX. Curr Protoc Bioinf Chap 2:Unit. 10.1002/0471250953.bi0203s00. 2.3 10.1002/0471250953.bi0203s00 18792934

[CR43] Waghorn TS, Knight JS, Leathwick DM (2014) The distribution and anthelmintic resistance status of *Trichostrongylus colubriformis*, *T. vitrinus* and *T. axei* in lambs in New Zealand. N Z Vet J 62:152–159. 10.1080/00480169.2013.871193 24313262 10.1080/00480169.2013.871193

[CR44] Wattanawong O, Iamsirithaworn S, Kophachon T, Nak-Ai W, Wisetmora A, Wongsaroj T, Dekumyoy P, Nithikathkul C, Suwannatrai AT, Sripa B (2021) Current status of helminthiases in Thailand: A cross-sectional, nationwide survey, 2019. Acta Trop 223:106082. 10.1016/j.actatropica.2021.106082 34364893 10.1016/j.actatropica.2021.106082

[CR45] Watthanakulpanich D, Pongvongsa T, Sanguankiat S, Nuamtanong S, Maipanich W, Yoonuan T, Phuphisut O, Boupha B, Moji K, Sato M, Waikagul J (2013) Prevalence and clinical aspects of human *Trichostrongylus colubriformis* infection in Lao PDR. Acta Trop 126:37–42. 10.1016/j.actatropica.2013.01.002 23318934 10.1016/j.actatropica.2013.01.002

[CR46] Winkelhagen A, Mank T, de Vries P, Soetekouw R (2012) Apparent triclabendazole-resistant human *Fasciola hepatica* infection, the Netherlands. Emerg Infect Dis 18:1028–1029. 10.3201/eid1806.120302 22607719 10.3201/eid1806.120302 PMC3358177

