# Supplementary material for: Genotypic evidence of benzimidazole resistance and population structure insights in zoonotic Trichostrongylus colubriformis from Thailand
Source: Parasitol Res. 2026 Jun 15;125(1):102. doi: 10.1007/s00436-026-08713-2 (PMC13500420; doi:10.1007/s00436-026-08713-2)
Supplement: Supplementary file 1 [file 436_2026_8713_MOESM2_ESM.docx]

Supplementary file 2: Example of the susceptible and resistant allele amplicons obtained after gel electrophoresis
